# Unusual Cause of Small Bowel Perforation: A Case Report

**DOI:** 10.7759/cureus.28853

**Published:** 2022-09-06

**Authors:** Ohud T Alharbi, Muhammad A Saeed, Raneem H Alzaghran, Zakiyah S Almutairi, Fatimah K Almeathem

**Affiliations:** 1 Department of General Surgery, College of Medicine, Qassim University, Buraydah, SAU; 2 Department of General Surgery, King Saud Hospital, Unayzah, SAU; 3 College of Medicine, Qassim University, Buraydah, SAU

**Keywords:** fishbone, emergency, surgical case reports, foreign body, small bowel perforation

## Abstract

Ingesting foreign bodies in the GI tract is not common, especially among adults. Here, we present a case of a 38-year-old male with a hyper-dense linear foreign body perforating the distal ileum, which turned out to be a 5-cm long fish bone eaten about a month before the presenting symptoms.

## Introduction

Most foreign body ingestions in adults pass spontaneously; however, in patients with hernial sac, Meckel's diverticulum, inflammatory bowel disease/colitis, or mesenteric ischemia, they pose an increased risk of perforations as a direct consequence of ingesting a foreign substance. A study reported that a 13-cm synthetic esophageal tube had passed through a patient's stomach with advanced gastric cancer and esophageal stricture. The esophageal tube had passed through the entire GI tract without perforation or impaction [1]. Therefore, not all ingested foreign bodies are affected by the esophageal cricopharyngeal sphincter; most of all foreign bodies that reach the stomach pass through the intestine, with only a low percentage causing damage and serious consequences [2]. Here we are highlighting a case of distal ileum small bowel perforation caused by an ingested fishbone in a healthy patient with no intestinal disease or history of abdominal surgery.

## Case presentation

**Figure 1:**
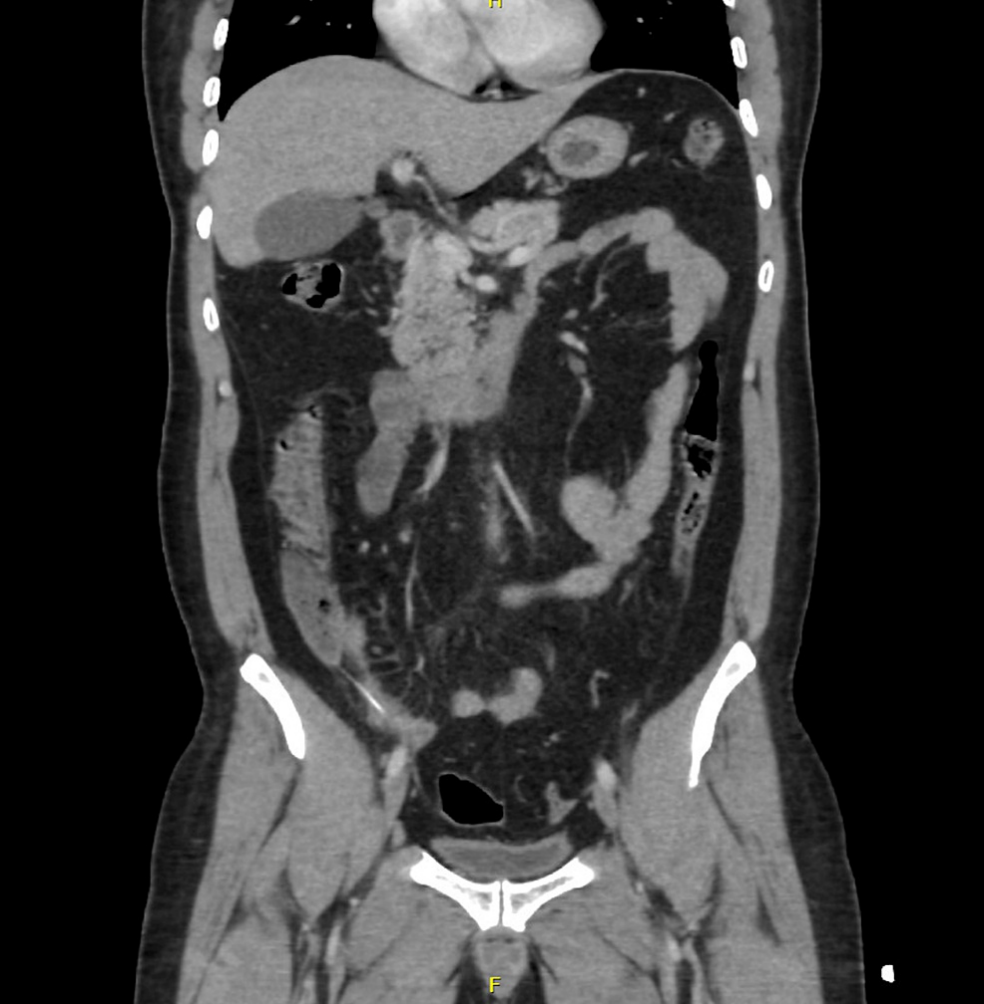
Coronal view of the foreign body in the distal ileum.

A 38-year-old man presented with acute abdominal pain that started 12 hours before admission. The pain was associated with nausea, vomiting, and diarrhea. During the examination, there was tenderness and rebound tenderness in the right iliac fossa and guarding. The laboratory tests revealed a WBC count of 13100/mm3 with 73.9% neutrophils. The chest and abdominal X-rays were unremarkable. Our preliminary diagnosis was acute appendicitis based on the clinical evaluation. A non-enhanced CT imaging study revealed a linear hyperdense structure, most likely a foreign body penetrating the distal ileum. There was no free fluid, pneumoperitoneum, or fat stranding in the vicinity (Figure 1).

**Figure 2:**
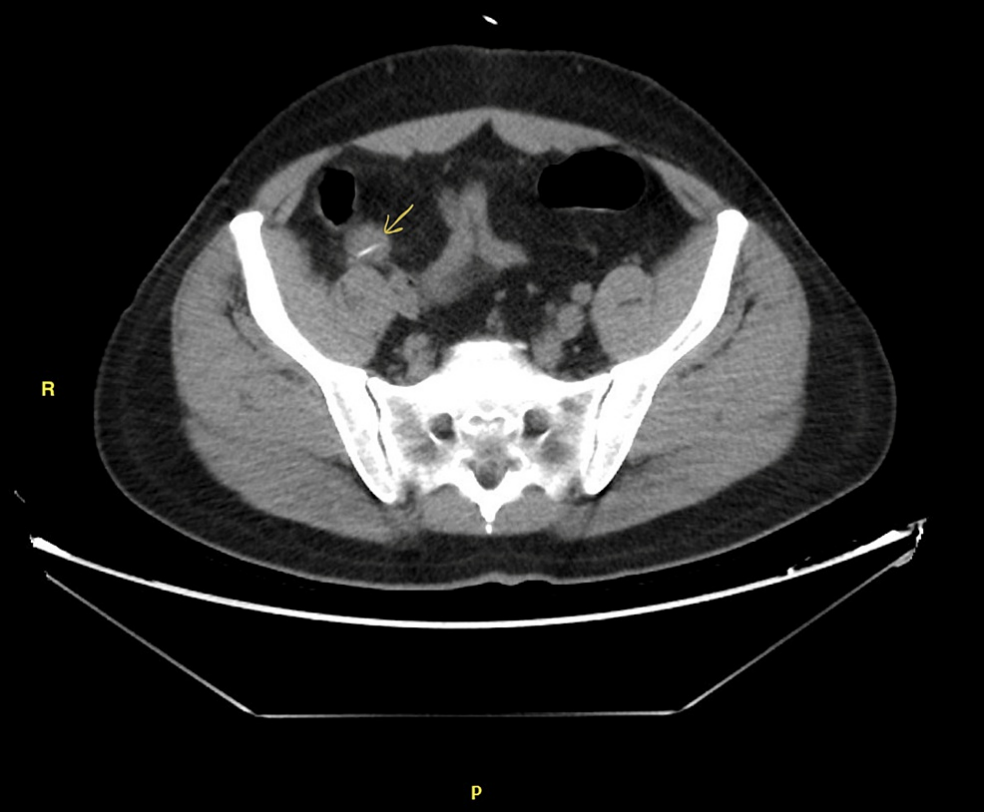
Axial view of the foreign body in distal ileum (yellow arrow).

A laparoscopy was performed on the patient. There was a 7-cm long, pointed foreign body perforating the distal ileum with no spillage of bowel contents. Half of the foreign body (fishbone) was still inside the bowel lumen (Figure 2).

**Figure 3:**
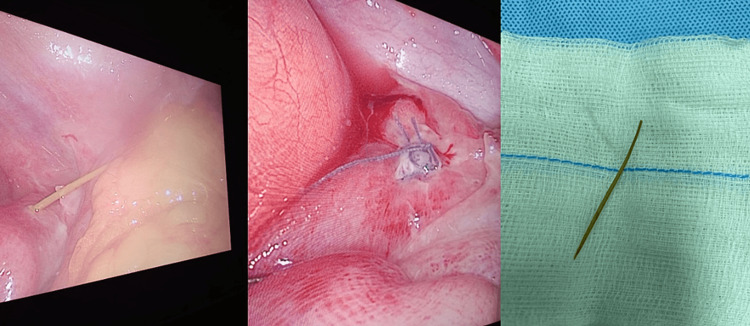
A) Fishbone emerging through the distal ileum during the surgery. B) Closed perforation using Lembert suture. C) The 5 cm long fishbone.

The fish bone was removed, and the perforation was closed with two 3/0 Vicryl Lembert sutures (Figure 3). No drains were placed. The whole procedure was completed laparoscopically. Post-operative recovery was uneventful. After eight hours, the patient was started on clear liquids and discharged the first day postoperatively.

## Discussion

In less than 1% of cases, foreign bodies in the intestine can cause intestinal perforation [1]. The bowel has a strong ability to protect itself, as its walls expand the lumen of the bowel at the point of contact, allowing freer passage to the offending object [3]. Furthermore, the bowel wall relaxes when swallowing something sharp and long, causing the head to point and the sharp end to trail behind [4]. However, more than 90% of swallowed foreign bodies pass through the gut if they reach the stomach [1]. Perforation occurs when a foreign body strikes and gradually erodes the intestinal wall. The perforation site is then covered by fibrin, omentum, or the bowel loops closest to it [5]. Foreign bodies may perforate through the GI tract via a hernial sac, Meckel's diverticulum, or the appendix. The terminal ileum, with its deep transverse rugae and sharp angulations, is a common site for the entrapment of long and thin objects, making it the most common perforation site, followed by the duodenal C-loop because of its rigidity and immobility. The vast majority of ingested foreign bodies pass through the GI tract unharmed, with only a few cases requiring surgical intervention [1,6]. The most frequent foreign object that results in GI tract perforations when accidentally swallowed are fish bones or chicken bone with sharp ends [1]. In our case, the pointed edge of a fish bone appears to be the cause of perforation. GI tract perforation is a severe condition; failure to recognize and treat it can lead to high mortality and morbidity rates. It has varied presentations, such as acute or chronic abdominal pain, GI bleeding, and bowel obstructions. Rarely unusual manifestations, such as ureteric colic, have also been reported. [7]. Bowel perforation secondary to a foreign body presents as an acute abdominal emergency. Patients often have severe tenderness, rebound tenderness, nausea, vomiting, and fever after perforation. Nonetheless, detecting a foreign body-induced perforation prior to surgery is uncommon [8,9]. Diagnosis of a GI tract perforation caused by foreign bodies is incredibly hard, yet it is critical to add it to one's differentials whenever acute abdominal symptoms arise. In our case, the distal ileum was perforated.

## Conclusions

Patients who swallow a fish bone or sharp foreign body should undergo endoscopy as soon as possible to remove the foreign body and avoid non-traumatic bowel perforation. The presence of a history of ingesting a foreign body should raise a lot of concern and suspicion in the minds of clinicians. Retrieval of the fishbone by a minimally invasive procedure can result in reduced morbidity and hospital stay. Bowel perforation should always be one of the differential diagnoses of acute abdomen.
